# Transcriptome Analysis of Quantitative Resistance-Specific Response upon *Ralstonia solanacearum* Infection in Tomato

**DOI:** 10.1371/journal.pone.0046763

**Published:** 2012-10-05

**Authors:** Takeaki Ishihara, Ichiro Mitsuhara, Hideki Takahashi, Kazuhiro Nakaho

**Affiliations:** 1 Plant Protection Division, National Agricultural Research Center, National Agriculture and Food Research Organization, Tsukuba, Ibaraki, Japan; 2 Division of Plant Sciences, National Institute of Agrobiological Sciences, Tsukuba, Ibaraki, Japan; 3 Department of Life Science, Graduate School of Agricultural Science, Tohoku University, Sendai, Miyagi, Japan; Nanjing Agricultural University, China

## Abstract

Bacterial wilt, caused by the soil-borne bacterium *Ralstonia solanacearum*, is a lethal disease of tomato, but the molecular mechanisms of the host resistance responses to *R. solanacearum* remain unclear. In this study, we report the first work describing the transcriptome of cultivar resistance and susceptible tomato cultivar after inoculation with *R. solanacearum*. To elucidate the characteristics of resistance early in the interaction, we analyzed microarrays for resistant cultivar LS-89 and susceptible cultivar Ponderosa 1 day after stem inoculation. No change in gene expression was detected for Ponderosa, but expression levels of over 140 genes, including pathogenesis-related, hormone signaling and lignin biosynthesis genes, increased in LS-89. Expression of β-1,3-glucanase genes increased substantially. In an immunohistochemical study, glucanase in LS-89 accumulated in the xylem and pith tissues surrounding xylem vessels filled with *R. solanacearum*. The expression of these genes also increased in four other resistant cultivars, but changed little in four susceptible cultivars in response to *R. solanacearum*, suggesting that similar reactions occur in other cultivars. These gene expression profiles will serve as fundamental information to elucidate the molecular mechanisms in the resistance response to *R. solanacearum* in tomato.

## Introduction

Bacterial wilt caused by *Ralstonia solanacearum* is a major constraint in the production of solanaceous crops in tropical, subtropical and some warm temperate regions worldwide [1]. Control of the disease with chemicals and crop rotation is insufficient because the pathogen is particularly well adapted for surviving in the soil and rhizosphere. Therefore, cultivar resistance is the most effective means to control bacterial wilt in many crops, including tomato (*Solanum lycopersicum*) [1].

Tomato is one of the most important crops in the world, and numerous efforts to breed bacterial wilt-resistant cultivars have been undertaken for decades. In Japan and elsewhere, grafting of susceptible but high-quality tomato cultivars onto resistant rootstock cultivars has been widely adopted to manage bacterial wilt [2]. Major sources of bacterial wilt resistance in cultivated tomato are wild tomatoes *S. pimpinellifolium* and *S. lycopersicum* var. *cerasiforme*. For instance, highly resistant lines Hawaii7996 and Hawaii7998 are thought to have been derived from PI127805A (*S. pimpinellifolium*) [3], [4]. Even though a resistance response is induced in both roots and stems, resistance to bacterial wilt is not associated with inhibition of bacterial root invasion but with the ability of the plant to limit bacterial colonization in the stem; thus, the degree of disease resistance is related to the extent that *R. solanacearum* spreads in the stem tissues [5], [6]. The defense response to *R. solanacearum* in tomato is tolerance rather than immunity, and this resistance sometimes breaks down at high temperature, humidity or pathogen density [1]. Therefore, information on the defense mechanisms in the stem is essential for breeding cultivars with reliable bacterial wilt resistance or for effective use of grafting cultivation to increase host plant resistance.

Natural plant defenses against *R. solanacearum* have been genetically analyzed. In *Arabidopsis thaliana*, resistance to *R. solanacearum* strain GMI1000 in accession Nd-1 is inherited as a single recessive resistance gene, *RRS1-R* [7]–[9], and in accession S96, a single dominant locus for resistance to strain Ps95 is present [10]. In contrast, resistance in tomato is complex and controlled by several quantitative trait loci (QTLs). Studies examining the progeny of a cross between Hawaii7996 and a susceptible line revealed QTLs on chromosomes 3, 6, 8, 10 and 11 under growth chamber conditions and/or field conditions [11], [12]. Similar analysis of resistant cultivar L285 (*S. lycopersicum* var. *cerasiforme*) revealed QTLs on chromosome 6, 7 and 10 [13], [14]. Therefore, the bacterial wilt resistance in tomato is generally under polygenic control, while the control of resistance in *A. thaliana* is monogenic.

The histopathological characteristics of tomato plants infected with *R. solanacearum* are well documented. *Ralstonia solanacearum* requires only small wounds in the roots, such as those formed during lateral root emergence, to establish a systemic infection, which spreads rapidly throughout the vascular system and thereby suppressing water flux [15], [16]. In resistant tomato cultivars, physical barriers have been suggested to play important roles in preventing bacterial spread. In resistant cultivar Caraïbo, which has resistance derived from *S. lycopersicum* var. *cerasiforme* [3], many tyloses occlude colonized xylem vessels and adjacent vessels [17]. Modification of pectic polysaccharides in the cell wall was also noted in resistant cultivars and during induced resistance responses [18]–[20]. In LS-89 (a selection from Hawaii7998), a popular commercial resistant rootstock cultivar in Japan, bacteria were localized in the primary xylem tissues both in infected upper hypocotyls and stems, whereas in commercial susceptible cultivar Ponderosa, bacteria were found in both primary and secondary xylem tissues and often in intercellular spaces of necrotic cells in the xylem and nearby pith tissues [21]–[23]. Electron microscopic analysis revealed that the limitation of bacterial spread was associated with increased electron density and thickness of the pit membranes in vessels, with an accumulation of electron-dense materials around the pits, and with the development of apposition layers in parenchyma cells adjacent to the vessels with bacteria [24]. Interestingly, these histopathological changes in LS-89 were significantly reduced and delayed after inoculation with a pectinase-deficient mutant of *R. solanacearum* [25]. Pectinases of *R. solanacearum* might play a role in eliciting structural changes, possibly through the release of oligogalacturonides that trigger plant defenses. Others have suggested that pectinases have a role in the resistance reaction [18]–[20]. However, the molecular mechanisms underlying the induction of defense responses in resistant tomato cultivars remain unclear.

In general, ethylene (ET), jasmonic acid (JA) and salicylic acid (SA) function as important signaling molecules of defense responses in plants. ET/JA-mediated signaling pathways have been shown to play roles in defense responses, particularly against necrotrophic pathogens, while SA-regulated defense responses are effective against biotrophic pathogens, and there is an antagonism between the ET/JA-dependent and the SA-dependent pathways [26]. Recently, *Tobacco rattle virus*-based virus-induced gene silencing technique was used to analyze the involvement of ET, JA and SA in the defense response of resistant cultivar Hawaii7996 against *R. solanacearum* [27], [28]. Interestingly, silencing of the genes involved in ET (*ACO1/3*, *EIN2* and *ERF3*), JA (*COI1*) and SA (*NPR1*, *TGA2.2* and *TGA1a*) signaling resulted in significant increases in bacterial proliferation in stembases and/or mid-stems. These results suggested that the ET-, JA- and SA-dependent pathways are engaged in the defense response of tomato to *R. solanacearum*.

In addition to genetic analyses, differential proteomics studies have been reported. Afroz et al. [29] found that nine proteins were differentially expressed in healthy tomato plants of susceptible and resistant cultivars. After treatment with JA or SA, one of the nine proteins was significantly down-regulated in the JA-treated resistant cultivar but expression did not change in the JA-treated susceptible cultivar. Similarly, another of the nine proteins increased in the SA-treated susceptible cultivar but not in the SA-treated resistant cultivar. When the proteome level of the tomato mid-stem was analyzed 5 days post inoculation (dpi) with *R. solanacearum*, protein expression in the resistant line did not change even though 12 proteins were more abundant in the susceptible line [30]. In studies of cell wall proteins extracted from purified cell walls of resistant and susceptible stems at 5 dpi with *R. solanacearum*, the levels of seven proteins increased and eight decreased in resistant and those of five proteins increased and eight decreased in susceptible cultivars [31]. Among these, the levels of pathogenesis related (PR), other defense related and glycolytic proteins increased in both cultivars. However, information on the mechanisms underlying the defense responses in this host–parasite interaction is still limited, and whether the detected alterations in the proteins are part of a defense response or the result of infection is still unclear because susceptible cultivars had already developed symptoms by 4 dpi.

Because molecular information on *R. solanacearum* resistance is limited, global transcriptional analyses are indispensable to elucidate the characteristics of the defense responses. In a transcriptome analysis of the interaction between *A. thaliana* and *R. solanacearum* [32], only a few genes were preferentially expressed early in the resistance response. Further, it is unclear whether the molecular mechanisms of polygenic resistance in tomato are the same as for monogenic resistance in *A. thaliana*. In fact, several studies have revealed that the molecular mechanisms and response of tomato to pathogens may differ from those of *A. thaliana*; thus, data gained from the *A. thaliana* cannot be applied directly to the tomato [33], [34].

In the present study, we analyzed gene expression levels in the stems of a resistant cultivar, LS-89, and a susceptible cultivar, Ponderosa, at an early stage of their interaction and found that the expression levels of over 140 genes increased in LS-89, whereas no changes in gene expression were detected in Ponderosa. Expression data suggested that the ET and JA signaling pathways are involved in signal transduction, because of an increase in expression of *PR* genes such as those encoding β-1,3-glucanases and lignin and hydroxycinnamic acid amides (HCAAs), which might act as physical barriers to prevent bacterial movement and proliferation. The gene expression profiles that we report here highlight some of the characteristics of quantitative resistance to *R. solanacearum* in LS-89 and may become powerful tools for elucidating the molecular mechanisms of resistance responses to *R. solanacearum* in tomato.

## Results

### Bacterial Density in LS-89 and Ponderosa Infected with *R. solanacearum*

**Figure 1:**
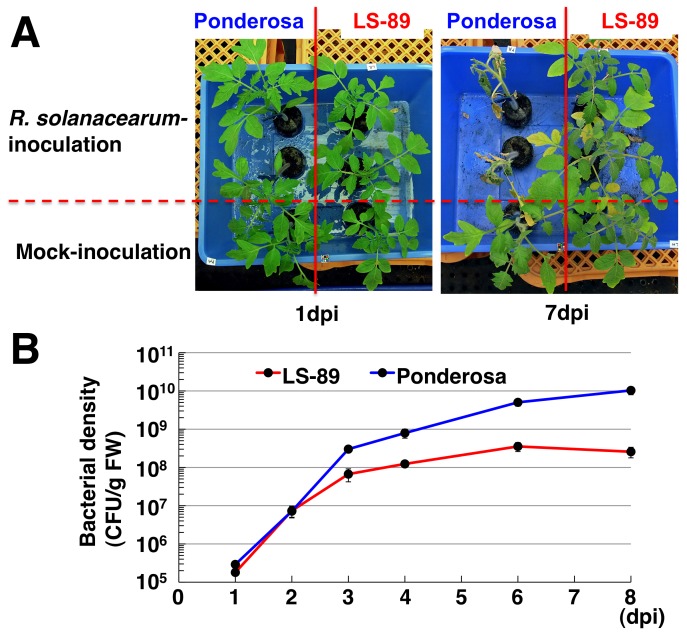
Symptoms on susceptible tomato cv. Ponderosa and resistant tomato cv. LS-89 after inoculation with *Ralstonia solanacearum* or water (mock) and bacterial density over time. (A) Symptoms at 1 and 7 dpi. (B) Bacterial density, determined on selective medium, in stems of four to eight randomly selected plants at 1, 2, 3, 4, 6 and 8 dpi.

We inoculated stems with a suspension of *R. solanacearum* (1.0×10^6^ colony forming units [CFU]/ml) just above the cotyledon of resistant cultivar LS-89, a popular commercial rootstock in Japan, and susceptible cultivar Ponderosa by cutting the stem to one-third of its diameter with a razor [22]. In susceptible Ponderosa, *R. solanacearum*-inoculated plants had no symptoms by 2 dpi, then started to wilt at 3 or 4 dpi and had wilted completely by 7 dpi. On the other hand, no symptoms were observed at 7 dpi in resistant LS-89 (Figure 1A), and they had not wilted even at 30 dpi (data not shown). Bacterial densities in the stem 5 mm below the inoculation site were the same at 1 and 2 dpi in both cultivars, but were lower in LS-89 than in Ponderosa at 3 dpi. The densities approached saturation at 6 to 8 dpi with a titer of about 10^8^ CFU/g fresh matter (FM) in LS-89 but with more than 10^9^ CFU/g FM in Ponderosa (Figure 1B). Therefore, we regarded 1 dpi as an early stage of interaction and an appropriate time point for analyzing expression profiles.

### Analysis of the Gene Expression Profiles of LS-89 and Ponderosa Infected with *R. solanacearum*

**Figure 2:**
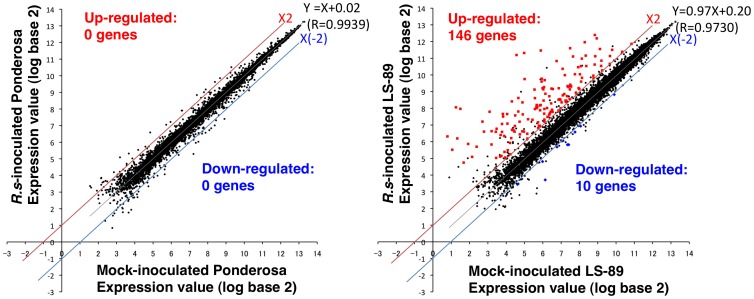
Scatter plots of mean logarithmic values for gene expression in LS-89 and Ponderosa at 1 dpi after inoculation with *Ralstonia solanacearum* or water (mock). In triplicate experiments using GeneChip, tomato probe sets with less than three ‘present’ calls were removed from the plot. Red squares: genes with significantly increased expression, blue diamonds: genes with significantly suppressed expression, closed circles: genes with unaltered expression. Gray lines: regression lines, red lines: 2-fold induction, blue lines: 2-fold suppression. Regression equation and correlation coefficient (*R*) are also shown.

For global gene expression analysis, LS-89 and Ponderosa were inoculated with *R. solanacearum* (1.0×10^6^ CFU/ml) or water as a control mock inoculation, and gene expression profiles at 1 dpi were analyzed using an Affymetrix Tomato Genome Array GeneChip representing over 9,200 tomato genes. The RNA from 15 plants at 1 dpi was used for each hybridization, and three biological replicates were performed. Within the six experiments (three biological replicates in mock- and *R. solanacearum*-inoculated), probe sets with less than three ‘present’ calls were removed from the statistical test. Figure 2 shows scattered groups displaying the average expression values in LS-89 and in Ponderosa infected with *R. solanacearum* on the *y*-axis and those in the mock-inoculated samples on the *x*-axis. The correlation coefficient (*R*) in LS-89 was slightly lower than in Ponderosa (*R* = 0.9730 in LS-89 and *R* = 0.9939 in Ponderosa), indicating that expression patterns between the inoculation with *R. solanacearum* and the mock-inoculation changed more in LS-89 than in Ponderosa. Meanwhile, the regression line approached *y* = *x* (*y* = 0.97*x*+0.20 for LS-89 and *y* = *x*+0.02 for Ponderosa), and *R* values were nearly 1 in both cultivars, showing that the expression levels of the majority of tomato genes were not altered. Significance analysis of microarrays (SAM) [35] was performed for each cultivar to identify any genes that were altered significantly in their expression profiles in response to *R. solanacearum* compared with the corresponding mock-inoculated sample. Subsequently, a *q* value was calculated for each gene to estimate the false discovery rate (FDR) [36]. A difference with a *P* value <0.01, *q* value <0.1 and fold change >2.0 was considered significant. In LS-89, 184 genes (164 increased expression, 20 decreased) had a *P* value <0.01 and fold change >2.0. Among these, 146 genes with increased and 10 with decreased expression were identified as significantly differentially expressed at the 10% FDR threshold (*q* value <0.1) (Table S1). In Ponderosa, even though five genes (four increased and one decreased) with *P* value <0.01 and fold change >2.0 were found, but their *q* values >0.1 and thus their expression was not considered to be significantly different. In Figure 2, differentially induced and suppressed genes are shown by red squares and blue diamonds, respectively.

**Figure 3:**
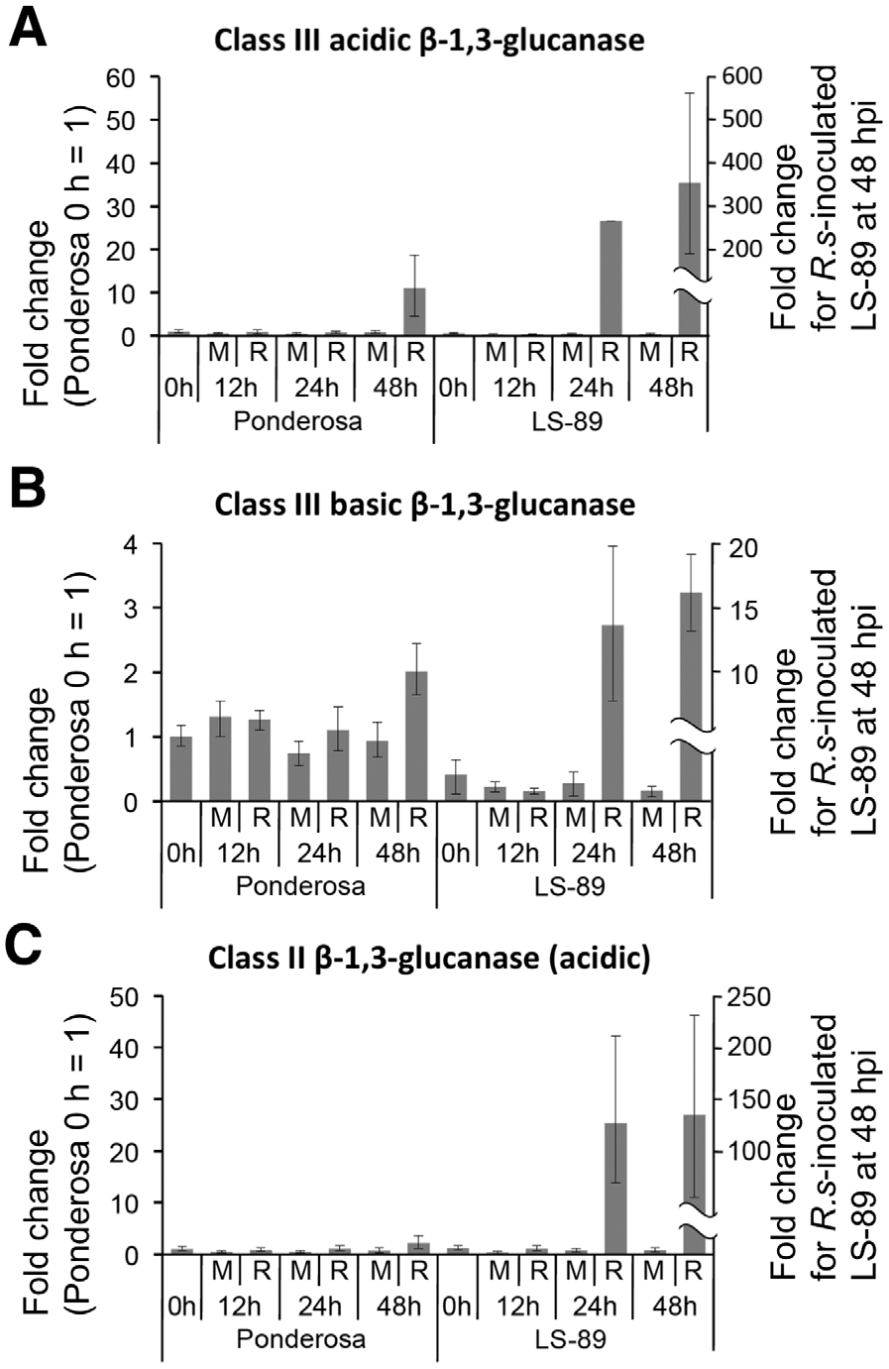
Real-time quantitative RT-PCR analysis of time course of relative transcription levels of β-1,3-glucanase genes in LS-89 and Ponderosa after inoculation with *Ralstonia solanacearum* or water (mock). Total RNA was extracted from stems to analyze expression of acidic class III (A), basic class III (B) and class II (C) β-1,3-glucanase genes at 0, 12, 24 and 48 hpi. Sample from Ponderosa at 0 hpi was used for calibration.

To validate the microarray results, we determined the transcript levels of the differentially expressed genes using a real-time RT-PCR analysis with specific primers for 13 up-regulated genes (three β-1,3-glucanase genes [Figure 3] and 10 genes including chitinases, *ACO1*, *Pti5* and WRKY transcription factors [Figure S1]). Consistent with the microarray results, the expression levels of all of these genes at 1 dpi were induced in LS-89 and were not induced or showed limited changes in Ponderosa.

### Characteristics of Genes with Altered Expression Levels

**Figure 4:**
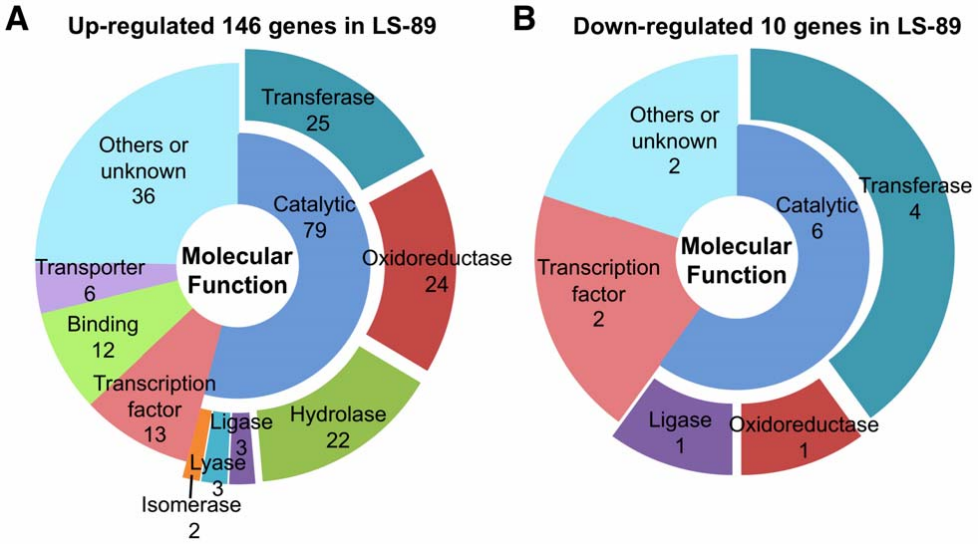
Molecular functional groups of genes that were differentially expressed in resistant tomato cultivar LS-89 after inoculation with *Ralstonia solanacearum*. Up- (A) and down- (B) regulated genes were classified according to Molecular Function in Gene Ontology. Genes with ‘catalytic activity’ were further subdivided into enzymatic function. Numbers indicate the number of genes found for each group.

Genes with altered expression levels in *R. solanacearum*-inoculated LS-89 and Ponderosa stems at 1 dpi were annotated using appropriate techniques including BLAST homology searches (http://blast.ncbi.nlm.nih.gov/) and the Gene Ontology (GO; http://www.geneontology.org/) (All genes with altered expression are listed in Table S1). Based on the information for Molecular Function in GO, the functions of the genes with altered expression were classified into broad functional groups: (1) catalytic activity (i.e., enzymatic activity), (2) transcription factor activity, (3) binding and (4) transporter activity, and genes with other activities and with unknown function were classified into (5) others or unknown (Figure 4 and Table S1). ‘Catalytic activity’ was further divided into narrower functional groups: ‘transferase activity’, ‘oxidoreductase activity’, ‘hydrolase activity’, ‘ligase activity’, ‘lyase activity’ and ‘isomerase activity’. Among the 146 up-regulated genes in LS-89, more than half of the genes (79 genes) were classified as ‘catalytic activity’ and nearly 10% (13 genes) were involved in ‘transcription factor activity’, which included five WRKY transcription factors (Figure 4A and Table S1). Similarly, among the 10 down-regulated genes in LS-89, more than half (six genes) belonged to the ‘catalytic activity’ class, and two were in the ‘transcription factor activity’ class (Figure 4B). Among the 79 genes in the ‘catalytic activity’ class in up-regulated genes in LS-89, major activities were ‘transferase activity’ (25 genes), ‘oxidoreductase activity’ (24 genes) and ‘hydrolase activity’ (22 genes) (Figure 4A).

Table 1 shows genes induced more than 10-fold in response to *R. solanacearum* in LS-89, including various disease response genes: *PR* genes, glutathione *S*-transferase, cytochrome P450 and Ca^2+^-signaling genes (calmodulin-binding family protein and calreticulin). The *PR* genes included *PR-5x*, *PR1b1*, *TSI-1* (*PR-10* family) and genes encoding hydrolases (i.e. β-1,3-glucanases and chitinases). ET, JA and auxin signaling genes were also induced more than 10-fold. Therefore, we collated differentially expressed genes involved in hormone signaling in Table 2. The gene with the second highest ratio of induced expression encoded lipid desaturase-like protein (Table 1 and 2) and was homologous to *A. thaliana FAD2*, which is involved in JA biosynthesis [37]. For auxin signaling, GH3 family indole-3-acetic acid (IAA)-amido synthetase gene was highly expressed (Table 1). In the JA and auxin signaling pathways, the expression of four genes encoding 3-ketoacyl-CoA thiolase, which is involved in the generation of JA and IAA, was induced (Table 2). The gene with the fifth highest fold-change encoded ACC oxidase 1 (ACO1) for ET biosynthesis (Table 1). In addition, the *ACS2* and β-cyanoalanine synthase genes, which are involved in ET biosynthesis, were induced in the resistance response (Table 2). Genes encoding ET responsive factor (ERF) transcription factors Pti5 and TSRF1 were also expressed (Table 2). In contrast, gibberellin (GA) signaling gene *GA20ox-3*, which is involved in the conversion of inactive forms of GAs into bioactive forms, was suppressed in LS-89 (Table 2).

**Table 1:** Genes induced more than 10-fold in response to *R. solanacearum* in resistant tomato cv. LS-89.

Probe Set ID	Description a	Fold change b	*P* c	*q* d
Les.3653.1.S1_at	Class III acidic β-1,3-glucanase (PR-Q’a)	111.7	0.0002	0.0173
Les.5934.1.S1_at	Lipid desaturase-like protein	99.0	0.0009	0.0414
Les.129.1.S1_at	Divinyl ether synthase (DES)	88.6	0.0012	0.0492
LesAffx.8850.1.S1_at	Esterase, putative	80.7	0.0010	0.0414
Les.2560.1.S1_at	ACC oxidase 1 (ACO1)	47.6	0.0002	0.0207
LesAffx.62420.1.S1_at	UDP-glucose:glucosyltransferase, putative	46.0	0.0022	0.0683
LesAffx.69808.1.S1_at	Calmodulin-binding family protein, putative	29.2	0.0003	0.0218
Les.3575.1.S1_at	DNA-binding protein Pti5	26.9	0.0002	0.0194
Les.3683.1.S1_at	PR-5 family member PR-5x	26.1	0.0001	0.0173
LesAffx.71065.1.S1_at	Pathogenesis-related family protein, putative	22.6	0.0003	0.0218
Les.5177.1.S1_at	Indole-3-acetic acid amido synthetase (GH3 family protein), putative	22.1	0.0003	0.0218
Les.3408.1.S1_at	PR (pathogenesis related) protein (PR1b1)	17.3	0.0024	0.0719
LesAffx.51300.1.S1_at	Unknown	17.2	0.0001	0.0173
Les.3652.1.S1_at	Class III basic β-1,3-glucanase (PR-Q’b)	16.9	0.0001	0.0173
LesAffx.11941.1.S1_at	Phytophthora-inhibited protease 1 (pip1)	15.5	0.0001	0.0173
LesAffx.66354.1.S1_at	Arogenate dehydrogenase, putative	15.4	0.0029	0.0825
Les.131.1.S1_at	Putative glutathione S-transferase T1	14.4	0.0002	0.0207
Les.37.1.S1_at	Class II chitinase (Chi2;1)	14.2	0.0001	0.0173
LesAffx.3059.1.S1_at	Transcription factor TSRF1	14.1	0.0002	0.0173
Les.4829.1.S1_at	2-Oxoglutarate-dependent dioxygenase (LeODD)	12.8	0.0001	0.0173
Les.1997.2.S1_at	Calreticulin, putative	12.7	0.0002	0.0173
Les.435.1.S1_at	Class III acidic chitinase, putative	12.7	0.0001	0.0173
LesAffx.52594.1.S1_at	Unknown	12.2	0.0004	0.0262
LesAffx.9038.3.S1_at	Cytochrome P450, putative	12.0	0.0001	0.0173
LesAffx.16164.1.S1_at	Calcium-binding EF hand family protein, putative	11.9	0.0002	0.0173
Les.4460.1.S1_at	Pathogenesis-related protein P2 (PR-P2)	11.7	0.0004	0.0247
Les.3635.1.S1_at	Subtilisin-like serine protease (P69B)	11.1	0.0002	0.0173
Les.3583.1.A1_at	TSI-1 protein (PR-10)	11.0	0.0013	0.0499
Les.3673.1.S1_at	Acidic extracellular β-1,3-glucanase (class II) (PR-2a)	10.8	0.0021	0.0671

a According to appropriate web tools including NCBI BLAST.

b Fold-change between mock- and *R. solanacearum*-inoculation, using fold-regulation cutoff of >2.0.

c Obtained using Significance Analysis of Microarrays (Tusher et al. 2001); *P*<0.01.

d Derived using method of Storey and Tibshirani (2003); *q* <0.1.

**Table 2:** Representative genes with altered expression levels in response to *R. solanacearum* in resistant tomato cv. LS-89 listed according to function.

Probe Set ID	Description a	Fold change b	*P* c	*q* d
**<Ethylene signaling>**				
Les.2560.1.S1_at	ACC oxidase 1 (ACO1)	47.6	0.0002	0.0207
Les.3575.1.S1_at	DNA-binding protein Pti5	26.9	0.0002	0.0194
LesAffx.3059.1.S1_at	Transcription factor TSRF1	14.1	0.0002	0.0173
Les.3662.1.S1_at	Ripening-related ACC synthase 2 (ACS2)	8.9	0.0007	0.0367
Les.3018.1.S1_at	Beta-cyanoalanine synthase, putative	2.0	0.0041	0.0999
**<Jasmonic acid signaling>**				
Les.5934.1.S1_at	Lipid desaturase-like protein	99.0	0.0009	0.0414
Les.129.1.S1_at	Divinyl ether synthase (DES)	88.6	0.0012	0.0492
Les.3493.1.S1_at	Phospholipase PLDb1	8.2	0.0001	0.0173
Les.3140.3.S1_at	Peroxisomal 3-ketoacyl-CoA thiolase, putative	3.5	0.0001	0.0173
Les.3140.2.S1_at	Peroxisomal 3-ketoacyl-CoA thiolase, putative	3.4	0.0002	0.0173
Les.2747.1.S1_at	Peroxisomal 3-ketoacyl-CoA thiolase, putative	3.2	0.0003	0.0240
Les.2747.2.S1_at	Peroxisomal 3-ketoacyl-CoA thiolase, putative	2.8	0.0002	0.0173
Les.3494.1.S1_at	Phospholipase PLDa2	2.0	0.0036	0.0954
**<Auxin signaling>**				
Les.5177.1.S1_at	Indole-3-acetic acid amido synthetase (GH3 family protein), putative	22.1	0.0003	0.0218
Les.2668.2.A1_at	Auxin and ethylene responsive GH3-like protein (GH3), putative	2.5	0.0009	0.0414
Les.3486.1.S1_at	Auxin-regulated protein	2.1	0.0035	0.0946
**<Giberellin signaling>**				
Les.63.1.S1_at	Gibberellin 20-oxidase-3 (GA20OX3)	-5.4	0.0013	0.0499
**<Carbohydrate metabolism>**				
Les.3653.1.S1_at	Class III acidic β-1,3-glucanase (PR-Q’a)	111.7	0.0002	0.0173
LesAffx.62420.1.S1_at	UDP-glucose:glucosyltransferase, putative	46.0	0.0022	0.0683
Les.3652.1.S1_at	Class III basic β-1,3-glucanase (PR-Q’b)	16.9	0.0001	0.0173
Les.37.1.S1_at	Class II chitinase (Chi2;1)	14.2	0.0001	0.0173
Les.435.1.S1_at	Class III acidic chitinase, putative	12.7	0.0001	0.0173
Les.4460.1.S1_at	Pathogenesis-related protein P2 (PR-P2)	11.7	0.0004	0.0247
Les.3673.1.S1_at	Acidic extracellular β-1,3-glucanase (class II) (PR-2a)	10.8	0.0021	0.0671
Les.122.1.S1_at	Class II chitinase	9.5	0.0001	0.0173
Les.3460.1.S1_at	Cell-wall invertase (Wiv-1)	9.2	0.0013	0.0499
Les.3777.1.S1_at	Wound-induced gene from tomato (twi1); glucosyltransferase,putative	5.8	0.0002	0.0173
Les.3779.1.S1_at	Class II acidic chitinase. Highly similar to *N.tabacum* PR-P and PR-Q	5.0	0.0003	0.0232
Les.3774.1.S1_at	Hexose transporter (HT2)	3.5	0.0006	0.0326
LesAffx.70524.1.S1_at	UDP-glucose:glucosyltransferase, putative	2.7	0.0010	0.0414
Les.5443.1.S1_at	Hydrolase, acting on glycosyl bonds/mannosyl-glycoproteinendo-beta-*N*-acetylglucosaminidase, putative	2.2	0.0004	0.0247
**<Lignin and/or HCAA biosynthesis>**				
Les.3741.1.S1_at	Sinapyl alcohol dehydrogenase or cinnamyl alcohol dehydrogenase,putative (CAD)	4.9	0.0010	0.0414
Les.3383.1.S1_at	4-Coumarate-CoA ligase, putative (4CL)	2.2	0.0002	0.0173
Les.4038.1.S1_at	*N*-hydroxycinnamoyl-CoA:tyramine *N*-hydroxycinnamoyl transferaseTHT1. (THT1-3 or THT1-4)	5.0	0.0014	0.0510
Les.3687.1.S1_at	*N*-hydroxycinnamoyl-CoA:tyramine *N*-hydroxycinnamoyl transferaseTHT7-1	3.8	0.0022	0.0683

a Determined using appropriate web tools including NCBI BLAST.

b Fold-change between mock- and *R. solanacearum*-inoculation, using fold-regulation cutoff of >2.0.

c Obtained using Significance Analysis of Microarrays (Tusher et al. 2001); *P*<0.01.

d Derived using method of Storey and Tibshirani (2003); *q* <0.1.

**Figure 5:**
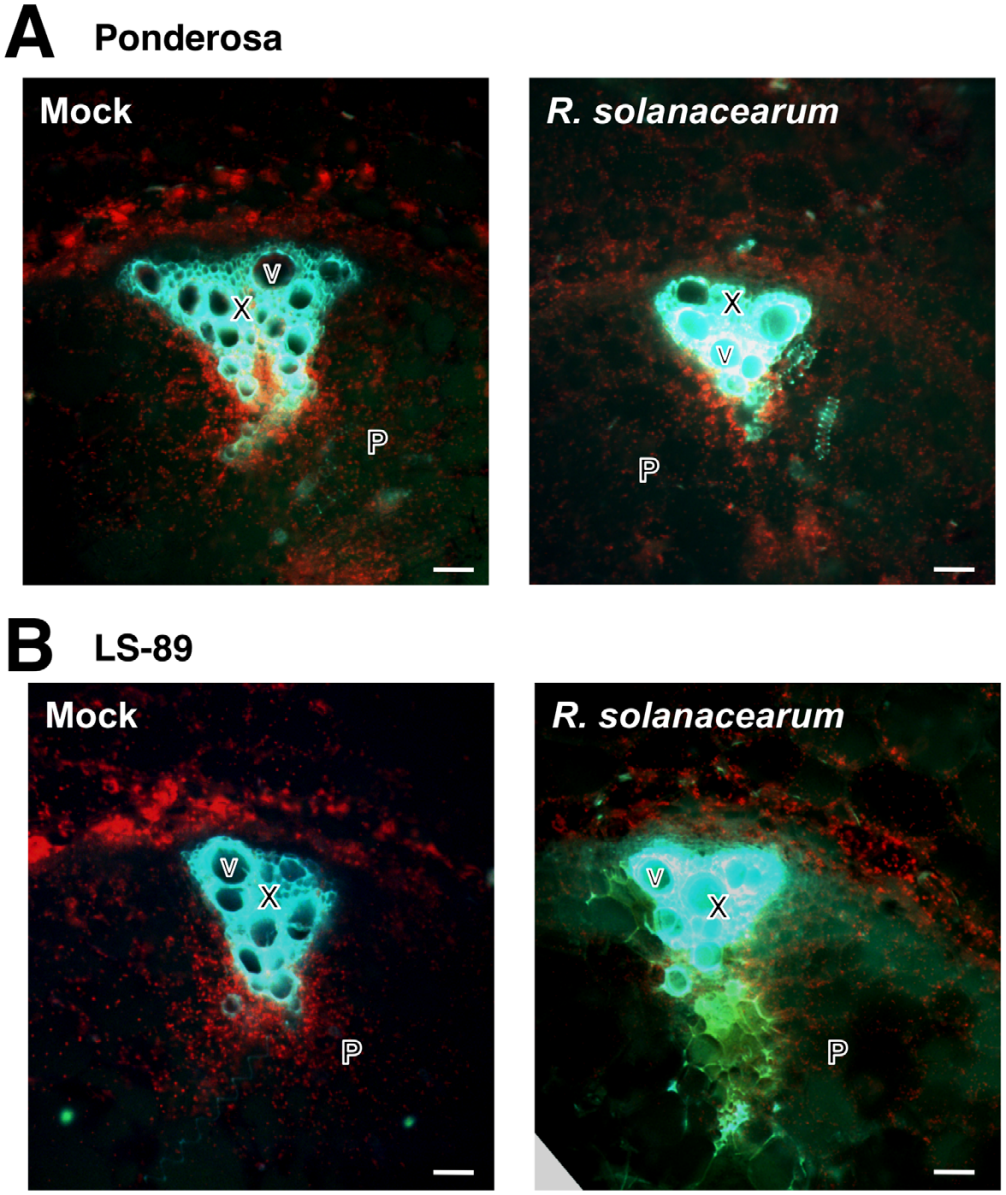
Comparison of autofluorescence of lignin in stem sections of susceptible tomato cv. Ponderosa (A) and resistant cv. LS-89 (B) after inoculation with water (mock) or *Ralstonia solanacearum*. In stems illuminated with UV and observed with a fluorescence microscope, green autofluorescence of xylem was present in all samples because lignin is naturally extremely high in xylem; the green autofluorescence of xylem in the pith increased only in the bacteria-inoculated LS-89 stems. Chloroplasts autofluoresce red. X, xylem; V, vessel; P, pith. Bar = 100 µm.

In addition to the hormone signaling genes, Table 2 includes genes with induced expression in LS-89 that are involved in carbohydrate metabolism and lignin and HCAA biosynthesis. For example, genes encoding β-1,3-glucanase, chitinase, cell wall invertase, which breaks down sucrose into glucose and fructose, hexose transporter and glucosyltransferase were induced (Table 2). Genes encoding 4-coumarate-CoA ligase (4CL) and cinnamyl alcohol dehydrogenase (CAD), which are involved in lignin biosynthesis, were induced in LS-89. In addition, two genes encoding hydroxycinnamoyl-CoA:tyramine *N*-hydroxycinnamoyl transferase (THT), which catalyzes the synthesis of HCAAs by conjugation of the intermediate product of lignin biosynthesis to β-phenylethylamine-alkaloids, were induced (Table 2). Because lignin compounds autofluorescence [38], we used fluorescence microscopy to analyze a cross section of the stem 5 mm below the inoculation site. Green autofluorescence of lignin in xylem was strong in all samples because of the naturally high levels in xylem. Consistent with the gene expression data, fluorescence intensity in the pith significantly increased in *R. solanacearum*-inoculated LS-89 stems, whereas this increase was limited in mock- or *R. solanacearum*-inoculated Ponderosa and mock-inoculated LS-89 stems (Figure 5).

### Time-course Expression Analysis

Class III acidic β-1,3-glucanase was the only gene for which expression increased over 100-fold, and the expression of class III basic and class II β-1,3-glucanase increased 16.9- and 10.8-fold, respectively (Table 1). Beta-1,3-glucanases have been subdivided based on protein isoelectric point and sequence similarity [39], [40]. In tomato, at least four β-1,3-glucanase proteins exist: one basic class I, one acidic class II, one acidic class III and one basic class III β-1,3-glucanases [40], [41]. Among these, the expression levels of the class II and the two class III genes were greatly induced in LS-89 (Table 1). Therefore, the expression of these three genes was analyzed in detail using real-time RT-PCR.

First, transcription levels of the two class III and the class II β-1,3-glucanase genes in mock- and *R. solanacearum*-inoculated LS-89 and Ponderosa stems were analyzed at 0, 12, 24 and 48 h post inoculation (hpi). Expression levels relative to the level in Ponderosa at 0 hpi are shown in Figure 3. In LS-89, these genes were not induced until 24 hpi. Their expression increased further at 48 hpi, 10-fold higher for the class III acidic β-1,3-glucanase and 5-fold higher for the class III basic and the class II β-1,3-glucanases over levels at 24 hpi. On the other hand, in Ponderosa, the class III acidic β-1,3-glucanase gene was induced only at 48 hpi, but its expression was much lower than in *R. solanacearum*-inoculated LS-89 stems at 24 hpi. Changes in expression levels of the class III basic and the class II β-1,3-glucanase genes in Ponderosa were limited throughout the study period.

In the time-course expression analysis of 10 other genes (Figure S1), expressions of all tested genes in *R. solanacearum*-inoculated LS-89 were clearly induced at 24 and 48 hpi. Interestingly, expression of two WRKY transcription factor genes had already been induced expression at 12 hpi in LS-89. In Ponderosa, the expression of Phytophthora-inhibited protease 1 (*pip1*) and LesAffx.51300.1.S1_at rarely changed, and that of two chitinases and LesAffx.837.1.S1_at (WRKY gene) was lower at 48 hpi than in LS-89. In contrast, the expression of *ACO1*, *Pti5*, 2-oxoflutarate-dependent dioxygenase (*LeODD*), Les.5443.1.S1_at and LesAffx.735.1.S1_at (WRKY gene) was high at 48 hpi. In particular, the expression of *LeODD* was much higher than in LS-89.

### Immunohistochemical Detection of Glucanase

**Figure 6:**
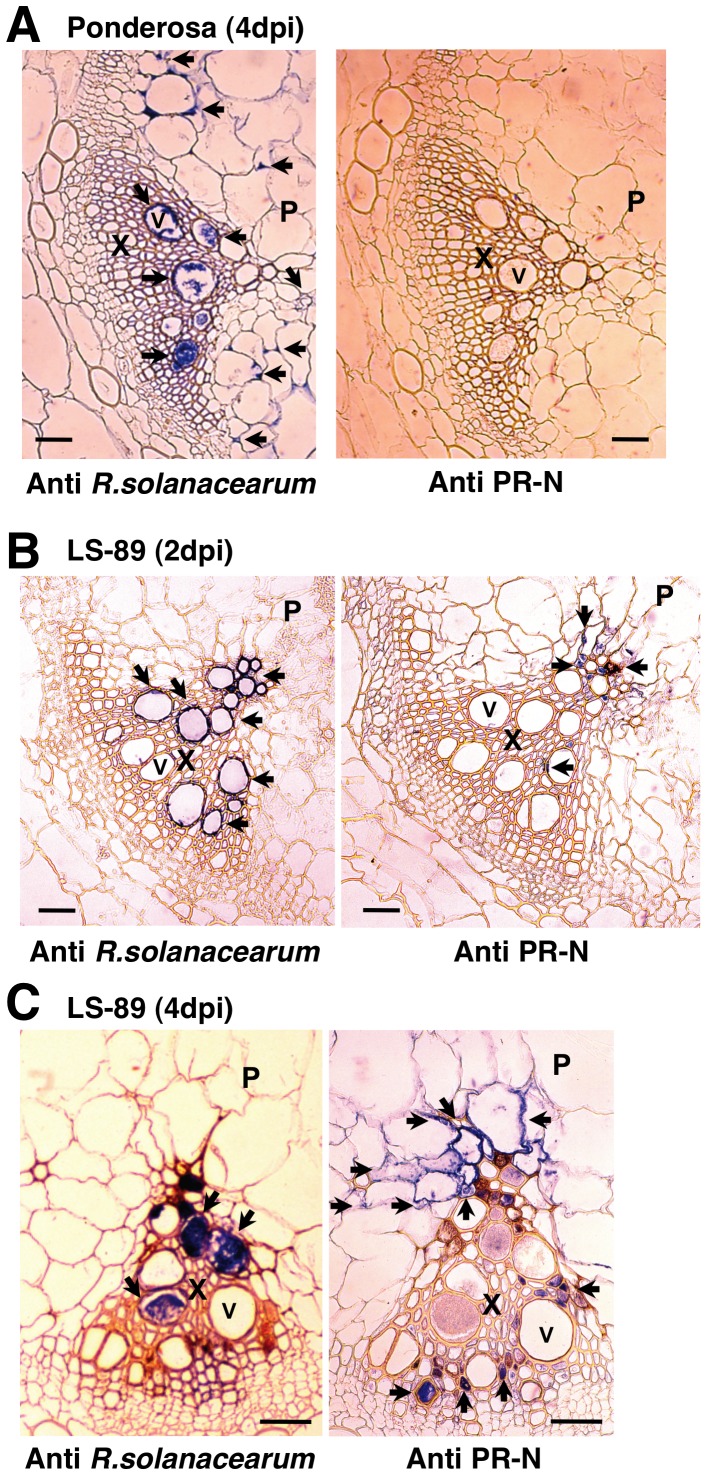
Immunohistochemical detection of *Ralstonia solanacearum* or tobacco PR-N protein, a class II acidic β-1,3-glucanase, in stem sections from susceptible tomato cv. Ponderosa at 4 dpi (A) and resistant cv. LS-89 at 2 dpi (B) and 4 dpi (C) after inoculation with *R. solanacearum*. Sections were exposed to antibodies against the bacteria or the protein, then stained with a VECTASTAIN ABC kit to localize the antibodies seen as blue signals (arrows). X, xylem; V, vessel; P, pith. Bar = 100 µm.

The spatial distribution of β-1,3-glucanase was investigated by immunohistochemical analysis using polyclonal antibodies against *R. solanacearum* [42] and tobacco PR-N, which is a class II acidic β-1,3-glucanase [43]. Class II and class III β-1,3-glucanases of tomato were shown to cross-react with the antiserum raised against PR-O, a class II β-1,3-glucanase from tobacco [44]. Thus, class II and class III β-1,3-glucanases share common antigenic sites, and the polyclonal antibody against tobacco PR-N used in this paper could recognize both the tobacco and tomato β-1,3-glucanase proteins. In the inoculated Ponderosa stems at 4 dpi, the blue signal of *R. solanacearum* was detected in the xylem and pith tissues, but a signal for β-1,3-glucanase was not detected (Figure 6A). In LS-89, *R. solanacearum* was detected only in xylem vessels at 2 and 4 dpi (Figure 6B and 6C). The blue signal of β-1,3-glucanase was not detected at 1 dpi (data not shown), but at 2 dpi, glucanase had slightly accumulated in the xylem and pith in the vicinity of *R. solanacearum* localization (Figure 6B). At 4 dpi, the glucanase signal had spread in the xylem and pith tissues surrounding the bacteria in the xylem vessels (Figure 6C).

### Expression Analysis in Other Cultivars

**Figure 7:**
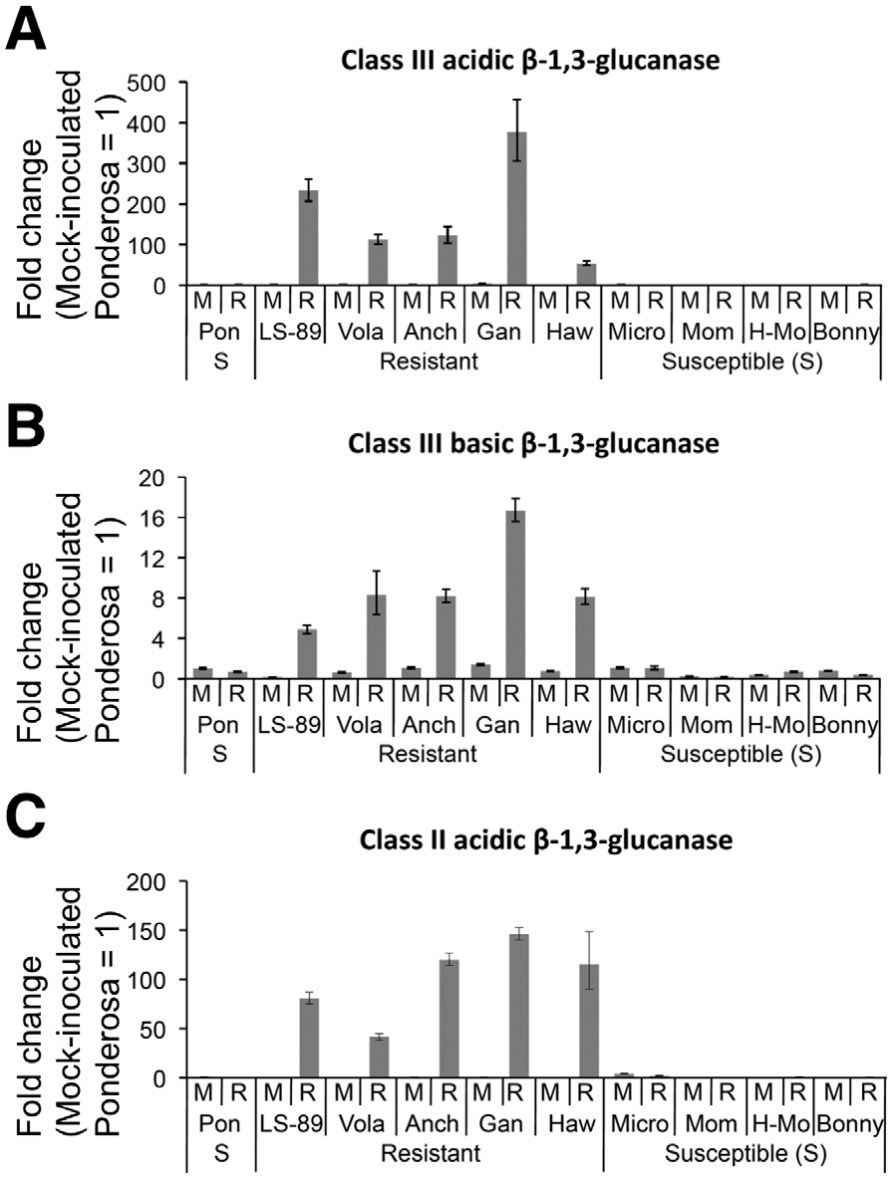
Real-time quantitative RT-PCR of relative transcription levels of β-1,3-glucanase genes in resistant and susceptible tomato cultivars at 1 dpi with water (mock, M) or *Ralstonia solanacearum* (R). Acidic class III (A), basic class III (B) and class II (C) β-1,3-glucanase genes were analyzed in total RNA from stems of five resistant cultivars LS-89, Volante (Vola), Anchor T (Anch), Ganbarune (Gan) and Hawaii7996 (Haw) and five susceptible cultivars (S) Ponderosa (Pon), Micro-Tom (Micro), Momotaro (Mom), House-Momotaro (H-Mo) and Bonny Best (Bonny). Sample from mock-inoculated Ponderosa stems was used for calibration.

We examined the expression of class III acidic, class III basic and class II β-1,3-glucanases at 1 dpi in four other resistant cultivars (Hawaii7996, Volante, Anchor T and Ganbarune) and four other susceptible cultivars (Bonny Best, Micro-Tom, Momotaro and House-Momotaro). Expression levels relative to the level in mock-inoculated Ponderosa stems are shown in Figure 7. The expression of these genes increased in all resistant cultivars, but changed very little in the susceptible cultivars in response to *R. solanacearum*. In the same analysis of 10 other genes (Figure S2), we obtained similar results for nine of genes, including *ACO1*, *Pti5* and chitinases. Expression of LesAffx.837.1.S1_at (WRKY gene) was also induced in LS-89 and in three other cultivars but was unchanged in resistant cultivar Volante and up-regulated in susceptible cultivars Momotaro, House-Momotaro and Bonny Best at 1 dpi. These differences might be due to natural variation in the cultivars’ timing of responses to pathogen infection.

## Discussion

Tomato bacterial wilt is a devastating disease caused by a soil-borne bacterium *R. solanacearum*, and elucidating the molecular mechanisms of resistant cultivars is very important. Although the transciptome of the silicon-induced resistance response of tomato to *R. solanacearum* was reported [45], the transcriptome of cultivar resistance was not. In this paper, we report the first transcriptome analysis in resistant and susceptible tomato cultivars using the Affymetrix GeneChip, representing over 9,200 tomato genes to elucidate defense mechanisms. We found that over 140 genes with various functions increased in resistant cultivar LS-89 at 1 dpi, whereas gene expression did not change in susceptible Ponderosa.

The degree of disease resistance in tomato is related to the spread of *R. solanacearum* in the stem tissues [5], [6]. In addition, Hikichi et al. [6] indicated that bacterial proliferation was suppressed in the stems below the first leaf of LS-89. Therefore, we targeted the stems for our global gene expression analysis of the defense response against *R. solanacearum*. Transcriptome analysis in the leaves of *A. thaliana* in response to root-inoculation with *R. solanacearum* has been reported [32]. Even though over 450 genes were differentially expressed between RRS1-R regulated resistance and compatible interactions at wilting phases (at 5 and 8 dpi), very few genes were differentially expressed in the early phases of the resistance response (at 6, 12 and 24 hpi). The absence of direct contact between the pathogen and the collected leaf samples could explain this observation. Further, simultaneous inoculation of the roots is technically difficult. Therefore, we considered that inoculating stems is the only way to monitor the early stage of resistance response in the stems. Introducing *R. solanacearum* directly into the stems has been exploited for decades to evaluate the resistance of tomato to bacterial wilt [46]. In our pathosystem, stem-inoculated Ponderosa started wilting at about 4 dpi and had completely wilted at 7 dpi, whereas stem-inoculated LS-89 had not wilted even at 30 dpi (Figure 1A and data not shown). Thus, the resistance response was normally induced in LS-89. Light microscopy of the inoculated stems revealed that bacterial masses were present only in the primary xylem tissues in LS-89, but they were found in both the xylem and pith tissues of Ponderosa (Figure 6) [22]. Moreover, a preliminary electron microscopic analysis of the inoculated LS-89 stems revealed increased electron density of the pit membranes in vessels, with accumulation of electron-dense materials around the pits and development of apposition layers in parenchyma cells adjacent to the vessels (data not shown). These results obtained by stem inoculation with LS-89 and Ponderosa were generally consistent with those after root inoculation in our previous reports [21], [23]–[25]. Therefore, it is possible that the characteristics of gene expression patterns in this paper are basically the same as those after natural infection of the roots.

Because the bacterial density in LS-89 and in Ponderosa first differed at 3 dpi (Figure 1B), resistance responses in LS-89 were induced earlier than 3 dpi. In fact, the global gene expression analysis revealed that the expression patterns of LS-89 and Ponderosa at 1 dpi were completely different (Figures 2 and 4). Furthermore, the expression of class III acidic β-1,3-glucanase gene was induced specifically in inoculated LS-89 stems at 1 dpi but was also induced in inoculated Ponderosa stems at 2 dpi, albeit at a lower level (Figure 3). These results suggest that gene expressions induced during a compatible interaction as well as during an incompatible interaction have already begun by 2 dpi. Ghareeb et al. showed a peak in resistance gene expression in *R. solanacearum*-inoculated tomato at 3 dpi, but found no early response [45]. The differences between our results and theirs might be due to differences in inoculation techniques and/or cultivars. We previously found that a low level but prompt response against infection in rice and blast fungus interactions greatly contributed to the suppression of the fungus [47]. We also observed in the interaction between *A. thaliana* and *Cucumber mosaic virus* that specific gene expression was induced much earlier than the appearance of hypersensitive cell death or disease symptoms [48]. Therefore, 1 dpi was an appropriate time to analyze global gene expression levels of resistance response in our system.

### Plant Hormone Signaling

Microarray data in this paper suggest that hormone signaling is involved in the defense response. In the ET biosynthetic pathway, ACS and ACO are key enzymes and catalyze the reaction from *S*-adenosyl-l-methionine to ACC and from ACC to ET, hydrogen cyanide (HCN) and CO_2_, respectively. Then, HCN is metabolized by β-cyanoalanine synthase [49]. The expression levels of *ACS2*, *ACO1* and β-cyanoalanine synthase gene were increased in response to *R. solanacearum* in LS-89. Consistent with these results is the report of Chen et al. [27] that the silencing of genes involved in ET signaling transduction pathways including *ACO1* caused breakdown of quantitative resistance against *R. solanacearum*. ERF transcription factor genes *Pti5* and *TSRF1* were also induced in LS-89 (Table 2). It is possible that they participate in activation of *PR* gene expressions [50], [51].

Transcriptome analysis also suggests that JA and auxin accumulate in LS-89 inoculated with *R. solanacearum*. A homolog of *A. thaliana FAD2*, which encodes lipid desaturase-like protein, was greatly induced in LS-89 (Table 1 and 2). FAD2 catalyzes the biosynthesis of linolenic acid in response to pathogen invasion, then linolenic acid is converted to hydroperoxides by lipoxygenase as a JA precursor [37]. Furthermore, the peroxisomal β-oxidation cycle, which plays a primarily role in fatty acid degradation and is involved in the generation of JA and IAA, may also be activated because the expression of four genes encoding 3-ketoacyl-CoA thiolase, which plays a major role in the β-oxidation cycle, was induced [52]. ET and JA signaling pathways have also been reported to be involved in silicon-induced resistance against *R. solanacearum* [45]. These results suggest that cultivar resistance and silicon-induced resistance share some common features.

### Characteristics of β-1,3-glucanase and Carbohydrate Metabolism

*Ralstonia solanacearum* in LS-89 was restricted to the xylem vessels, whereas the bacterium was detected in the xylem and pith tissues in Ponderosa (Figure 6). Thus, certain defense responses should be induced in the cells surrounding the xylem vessels. Considering that the β-1,3-glucanase was detected in the xylem and pith tissues surrounding the *R. solanacearum* detected in xylem vessels (Figure 6B and 6C), the defense response accompanied by β-1,3-glucanase accumulation may play an important role in preventing bacterial movement toward the outside of the xylem vessels. β-1,3-Glucanase was detected mainly in the extracellular spaces in LS-89 at 2 and 4 dpi (Figure 6B and 6C). Similar to our results, in an electron microscopic study using an antibody against tobacco PR-N, tomato glucanase accumulated predominantly in host cell walls and in secondary thickenings of xylem vessels [53]. In addition, both the tomato acidic and basic class III β-1,3-glucanases lack the C-terminal consensus sequence for sorting to the vacuole [41], and basic class III β-1,3-glucanase protein was detected in the xylem sap of tomato after infection with a fungal pathogen [54]. Class II β-1,3-glucanase protein has also been detected in apoplastic fluids [44]. These results suggest that glucanases encoded by the class II and the two class III β-1,3-glucanase genes are localized in the apoplast. Besides these, gene products of the induced *PR* genes including *PR-5x*, *PR1b1*, class II chitinase *Chi2;1* and *PR-P2* (Table 1) are localized in the extracellular spaces [54]–[56]. Involvement of the polysaccharide structure around the xylem vessels in resistance has also been well described, and pectic polysaccharides have been shown to contribute to physical barriers [18]–[20].

In the apoplast, perturbations of carbohydrate metabolism can occur because glucanase degrades β-glucan, chains of d-glucose polysaccharides, and the expression levels of other apoplastic carbohydrate metabolism genes, including apoplastic invertase and hexose transporter, which were also induced in the inoculated LS-89 stems (Table 2). Carbohydrate metabolism is frequently perturbed during infection. For instance, the accumulation of hexoses in the apoplast may act as an extracellular indicator of pathogen infection and induce defense systems [57]. In fact, overexpression of extracellular invertase genes leads to the accumulation of PR proteins [58]. Therefore, the apoplast may be an important site for defense responses to bacterial wilt in tomato stems.

Many WRKY transcription factors are involved in activation of PR genes [59], [60]. In our microarray analysis, among 13 up-regulated transcription factor genes, five encode putative WRKY type transcription factors (Table S1). The expression of two WRKY genes was induced earlier than β-1,3-glucanases, chitinases, *ACO1* and *Pti5* (Figure S1), suggesting that these WRKY transcription factors may play a key role in this resistance response. However, their functions and the pathways involved are still unclear. WRKY transcription factors belong to a large family, and the contribution of individual members to immunity is subtle because of their functional redundancy. Recently, tomato WRKY transcription factors SlWRKY72a, SlWRKY72b and SlWRKY70 were shown to contribute to a basal defense response and gene-for-gene resistance to root-knot nematode and potato aphid. Their functional aspects should differ because SlWRKY72 appears to control a SA-independent pathway, whereas SlWRKY70 contributes to a SA-dependent pathway [59], [60]. Future experiments should address the importance of WRKY transcription factors in the resistance response to *R. solanacearum* in tomato.

### Lignin and HCAA Biosynthesis

Lignin is primarily polymerized from hydroxycinnamyl alcohols (typically *p*-coumaryl alcohol, coniferyl alcohol and sinapyl alcohol), which are synthesized from phenylalanine by the activities of enzymes including 4CL and CAD [61]. In the inoculated LS-89 stems, the expression levels of the genes encoding 4CL and CAD were up-regulated (Table 2). Furthermore, we also observed autofluorescence, to which lignin contributes [38], specifically in the inoculated LS-89 stems (Figure 5). These results suggest that the interaction between LS-89 stem tissues and *R. solanacearum* induces lignin accumulation, probably as a physical barrier. Physical barriers can also be formed by pectic polysaccharides [18]–[20].

In addition, among intermediate products of lignin biosynthesis, hydroxycinnamic acids, such as *p*-coumaroyl-CoA and feruloyl-CoA, can occur conjugated to β-phenylethylamine-alkaloids, which are a group of nitrogenous, low molecular weight secondary metabolites, such as tyramine and octopamine, forming the corresponding HCAAs by the activity of THT. The expression of two THT genes was increased in our study. Increased expression of the THT gene preceded the accumulation of HCAAs [62]. Therefore, HCAAs may accumulate in *R. solanacearum*-inoculated LS-89 stems as they do in various incompatible interactions between tomato and bacterial pathogens [62]–[64]. HCAAs are thought to increase cell wall stability and decrease digestibility through peroxidative cross-linking in the cell wall, which leads to the formation of a barrier against pathogen ingress. Further, some HCAAs have antimicrobial activity. For example, HCAAs of dopamine [62] and tyramine [63] have antibacterial activity against *P. syringae* and *X. campestris*, respectively. These findings suggest that HCAAs may act directly and/or indirectly in defense against *R. solanacearum* infection.

### Conclusion

In this study, we obtained precise transcriptomic information on the early response of tomato against *R. solanacearum*. The expression of over 140 genes specifically increased in LS-89 stems. ET signaling and various other signaling pathways were coordinately involved in the exertion of resistance, which was followed by the accumulation of PR proteins, such as class III β-1,3-glucanases, mainly in the apoplast of the xylem and pith tissues. Lignin and HCAAs, which can function in physical barriers to prevent propagation of *R. solanacearum*, could play an important role in resistance. The characteristics of the expression patterns in this paper may not be specific to LS-89 and Ponderosa but shared by other resistant and susceptible cultivars because expression patterns of the β-1,3-glucanase genes and some other genes, including *ACO1*, *Pti5* and chitinase genes, at 1 dpi were similar in other cultivars tested, with minor exceptions (Figure 7 and Figure S2). Therefore, we believe the information obtained in this study offers clues to reveal detailed mechanisms of quantitative resistance to *R. solanacearum* in tomato plants and can be utilized in various ways, for instance for generating markers for the breeding of resistant cultivars.

## Materials and Methods

### Plant Growth

Bacterial wilt resistant tomato (*S. lycopersicum*) cultivars LS-89, Volante, Anchor T, Ganbarune and Hawaii7996 and susceptible cultivars Ponderosa, Micro-Tom, Momotaro, House-Momotaro and Bonny Best were used. Seedlings were raised individually in peat moss pellets (Jiffy-7, 43 mm single, Jiffy Products, Stange, Norway) and grown in a greenhouse at 25±5°C. About 30 days after sowing, seedlings at the five- to six-leaf stage were inoculated.

### Inoculation

*Ralstonia solanacearum* strain 8107S (race 1, biovar 4, phylotype 1) [21] was grown in selective TZC medium [65] containing 200 µg/ml streptomycin sulfate for 1 day at 30°C, with shaking. Bacteria were collected by centrifugation and resuspended in distilled water. The suspension was adjusted to 1.0×10^9^ CFU/ml by means of a colorimeter (OD600 = 1.00) (SmartSpec 3000 Spectrophotometer; Bio-Rad, Hercules, CA, USA), then diluted to 1.0×10^6^ CFU/ml. Stems of tomato seedlings were inoculated just above the cotyledon by cutting the stem to one-third of its diameter with a razor, adding 5 µl of bacterial suspension or distilled water for mock inoculation to the opening, then clipping the wound site to avoid bending [22]. Inoculated plants were grown in a growth chamber at 30°C under 30,000 lux light intensity for 12 h/day.

### Monitoring Bacterial Density in Plants

At 1, 2, 3, 4, 6 and 8 dpi, LS-89 and Ponderosa stems inoculated with 8107S strain were sampled by cutting round slices in 5-mm long sections at 5 mm below the inoculation site. Sections were weighed separately and homogenized using aluminum sticks in test tubes with 1 ml of distilled water. Each homogenate was serially diluted and plated onto selective medium amended with streptomycin. The plates were incubated at 30°C for 2 days. Colonies were counted, and the bacterial density (CFU/g FM) in the stem was calculated.

### Microarray Hybridization

At 1 dpi, LS-89 and Ponderosa stems that had been inoculated with 8107S strain or distilled water were sampled by dissecting 5-mm long sections at 5 mm below the inoculation site and were frozen immediately using liquid nitrogen. For each hybridization, RNA from 15 plants was isolated using an RNeasy Plant Mini Kit (Qiagen, Chatsworth, CA, USA) in accordance with the manufacturer’s protocol. Preparation of biotin-labeled probes from total RNA, hybridization with Affymetrix Tomato Genome Array GeneChip (Santa Clara, CA, USA), scanning and data collection were performed at an Affymetrix service provider (Kurabo Industries, Osaka, Japan). RNA quality and concentration were measured using an Agilent 2100 bioanalyzer (Agilent Technologies, Santa Clara, CA, USA), and 250 ng of total RNA was used for the following steps. Probe preparation, hybridization and scanning were performed in accordance with the One-cycle Target Labeling and GeneChip Expression Analysis Technical Manual, 701021 Rev. 5. The arrays were hybridized in a Hybridization Oven 640 (110 V) and washed and stained in Fluidics Station 450. Scanning was carried out with a GeneChip Scanner 3000 and image analysis was performed using GeneChip Operating Software ver1.4. Analysis of the data image and computation of the intensity for each cell was performed in accordance with the GeneChip Expression Analysis Data Analysis Fundamentals using the GeneChip Operating Software and MAS5 algorithm.

The expression data from this article have been deposited in the National Center for Biotechnology Information’s Gene Expression Omnibus (GEO) according to the MIAME guidelines and are accessible through GEO (accession no. GSE31807; http://www.ncbi.nlm.nih.gov/geo/query/acc.cgi?acc=GSE31807).

### Expression Profiling

Three biological replicates of microarray analysis were performed. The average of the signal intensity for each gene in three replicate experiments was used as the expression value of the gene. To select differentially expressed genes, we used the following criteria. Within the six experiments (three biological replicates in mock- and *R. solanacearum*-inoculated), probe sets with less than three ‘present’ calls were removed from the statistical test. *P* values were obtained using SAM [35], and statistical significance was set at *P*<0.01. To estimate the FDR, *q* values [36] were calculated, and probe sets having *q* >0.1 were excluded. Further, genes showing over a 2-fold change in the expression level in *R. solanacearum*-inoculated stems compared with mock inoculation were selected as differentially expressed. SAM and *q* value calculations were performed using the statistical software program R (ver. 2.13.2; R-project, Vienna, Austria; http://www.R-project.org/). The target sequences of the altered expression genes were obtained from the Affymetrix web site (http://www.affymetrix.com/analysis/index.affx). All genes with altered expression levels were annotated using the nucleotide BLAST program (http://blast.ncbi.nlm.nih.gov/) and Gene Ontology (http://www.geneontology.org/) with the homologous genes in *A. thaliana*, and other appropriate tools.

### Fluorescence Analysis of Lignification

UV autofluorescence from lignin was detected as described in López-Martín et al. [38]. At 4 dpi, stems (20 mm long) were sampled from the upper hypocotyls 5 mm below the inoculation site, and sections of the stem were cut using an NKsystem MTH-1 plant microtome (NKsystem, Osaka, Japan). The sections were then analyzed with a Nikon (Tokyo, Japan) MICROPHOTO-FXA optical microscope equipped with epifluorescence illumination (excitation filter UV-2A, 330–380 nm). All samples were photographed with the same magnification and exposure time.

### Real-time Quantitative PCR Expression Analysis

At 0, 12, 24 and 48 hpi, tomato cultivars inoculated with strain 8107S or distilled water were sampled by dissecting 5-mm long sections from 5 mm below the inoculation site. For each sample, total RNA from three plants was isolated using TRIzol reagent (Invitrogen, Carlsbad, CA, USA) in accordance with the manufacturer’s protocol. Isolated total RNA was treated with DNase I (Takara Bio, Shiga, Japan) to avoid contamination with genome DNA. After phenol–chloroform extraction and ethanol precipitation, the concentration of RNA was adjusted to 100 ng/µl of water using a spectrophotometer. Equivalent concentrations of the RNA samples were confirmed by electrophoresis. Then, total RNA was reverse transcribed using an iScript cDNA Synthesis Kit (Bio-Rad) in accordance with the manufacturer’s instructions; 5 µl (500 ng) of RNA was mixed with 2 µl of 5× iScript reaction mix, 0.5 µl of iScript reverse transcriptase and 2.5 µl of nuclease-free water and incubated at 25°C for 5 min, 42°C for 30 min and 85°C for 5 min. After the reaction, the sample was diluted with 190 µl of distilled water and used as a template DNA for real-time quantitative PCR. Real-time PCR was performed using SsoFast EvaGreen Supermix (Bio-Rad) and an Mx3000P Real-Time QPCR system (Stratagene, Santa Clara, CA, USA) in accordance with the manufacturers’ instructions with minor modifications. Primers were designed for class III acidic β-1,3-glucanase (TomQ’a-F, 5′-AAGCAAGAAGAGAGCATTAAAAGG-3′; TomQ’a-R, 5′-GTAATATGTTGGTTTCTTTATTAGCATATG-3′), class III basic β-1,3-glucanase (PRQb-F, 5′-ACGCGTTGTTTACATCCCCTGGA-3′; PRQb-R, 5′- AGTTGTTGTTGTAAGTCCTCGCGT-3′) and class II β-1,3-glucanase (A-glu-F, 5′- AACAGGAGCGCAGCCTATCGG-3′; A-glu-R, 5′- CCTTGGCGTTTGGAAGGATTGGC-3′). Tomato ubiquitin gene *UBI3* was used as a reference gene [66]. The primer sets used for Figures S1 and S2 are listed in Text S1. For the reaction, 2 µl of template DNA was mixed with 5 µl of SsoFast EvaGreen supermix, 0.06 µl each of forward and reverse primers (50 µM) and 2.88 µl of RNase/DNase-free water. PCR was performed using the following conditions: 95°C for 30 sec, one cycle; 93°C for 5 sec and 60°C for 10 sec (read cycle), 50 cycles. Specific amplification was confirmed by the generation of a dissociation curve. MxPro Software version 4.10 (Stratagene) was used to quantify the mRNA levels, with *UBI3* normalization by the ΔΔCt method.

### Immunohistochemical Analysis

At 1, 2 and 4 dpi, small blocks (5×5×5 mm) were excised from the upper hypocotyls (five each of the resistant and susceptible cultivars) at 5 mm below the inoculation site and fixed in a mixture of formalin, acetic acid, and 50% ethanol (1∶1:18 v/v/v) for 2 days or longer at 4°C. The fixed disks were dehydrated through a graded ethanol–xylene series, then embedded in paraffin. Transverse sections 15 to 20 µm thick were cut using a rotary microtome (PR-50; Yamato Koki, Tokyo, Japan). A Vectastain ABC-AP Kit (rabbit IgG) (Vector Laboratories, Burlingame, CA, USA) was used to immunohistochemically localize *R. solanacearum* and glucanase in tomato stem tissues as follows: (1) Sections were deparaffinized in xylene and rehydrated through a graded series of ethanol to water. (2) Deparaffinized sections were soaked for 15 min in 15% acetic acid to remove endogenous alkaline phosphatase (AP) activity, then washed twice (5 min each) in phosphate-buffered saline. (3) Sections were incubated for 20 min in blocking solution containing normal goat serum, then for 30 min in primary antibodies against *R. solanacearum* [42] or tobacco PR-N [43] (each diluted 1∶3000) in blocking solution. (4) Sections were washed, incubated with an AP-conjugated biotin-avidin complex, and developed chromogenically with Vector Blue (Vector Laboratories). Nonimmune normal serum was used instead of the primary antibody for a negative control. The sections were observed using a light microscope (BH-1; Olympus, Tokyo, Japan).

## Supporting Information

Figure S1 **Real-time quantitative RT-PCR analysis of time course of relative transcription levels of genes in resistant tomato cv. LS-89 and susceptible tomato cv.** **Ponderosa after inoculation with** ***Ralstonia solanacearum*** **(R) or water (mock, M).** Genes: ACC oxidase 1 (ACO1), DNA binding protein Pti5, two chitinases, phytophthora-inhibited protease1 (pip1), 2-oxoglutarate-dependent dioxygenase (LeODD), unknown protein (LesAffx.51300.1.S1_at), hydrolase (Les.5443.1.S1_at) and two WRKY transcription factors. Total RNA was extracted from stems to analyze expression at 0, 12, 24 and 48 hpi. Sample from Ponderosa at 0 hpi was used for calibration.
(TIF)

Figure S2 **Real-time quantitative RT-PCR to assess relative transcription levels of genes in stems of various tomato cultivars at 1 dpi with water (mock, M) or** ***Ralstonia solanacearum*** **(R).** Genes: ACC oxidase 1 (ACO1), DNA binding protein Pti5, two chitinases, phytophthora-inhibited protease1 (pip1), 2-oxoglutarate-dependent dioxygenase (LeODD), unknown protein (LesAffx.51300.1.S1_at), hydrolase (Les.5443.1.S1_at) and two WRKY transcription factors. Resistant cultivars: LS-89, Volante (Vola), Anchor T (Anch), Ganbarune (Gan) and Hawaii7996 (Haw). Susceptible cultivars: Ponderosa (Pon), Micro-Tom (Micro), Momotaro (Mom), House-Momotaro (H-Mo) and Bonny Best (Bonny). Sample from mock-inoculated Ponderosa stems was used for calibration.
(TIF)

Table S1 **Genes with altered expression levels in response to** ***R. solanacearum*** **in resistant tomato cv. LS-89.**
(XLS)

Text S1 **Primer pairs for real-time quantitative RT-PCR in Figures S1 and S2.**
(DOC)
