# Supplementary figures and images for: Transcriptome Analysis of Quantitative Resistance-Specific Response upon Ralstonia solanacearum Infection in Tomato
Source: PLoS One. 2012 Oct 5;7(10):e46763. doi: 10.1371/journal.pone.0046763 (PMC3465262; doi:10.1371/journal.pone.0046763)

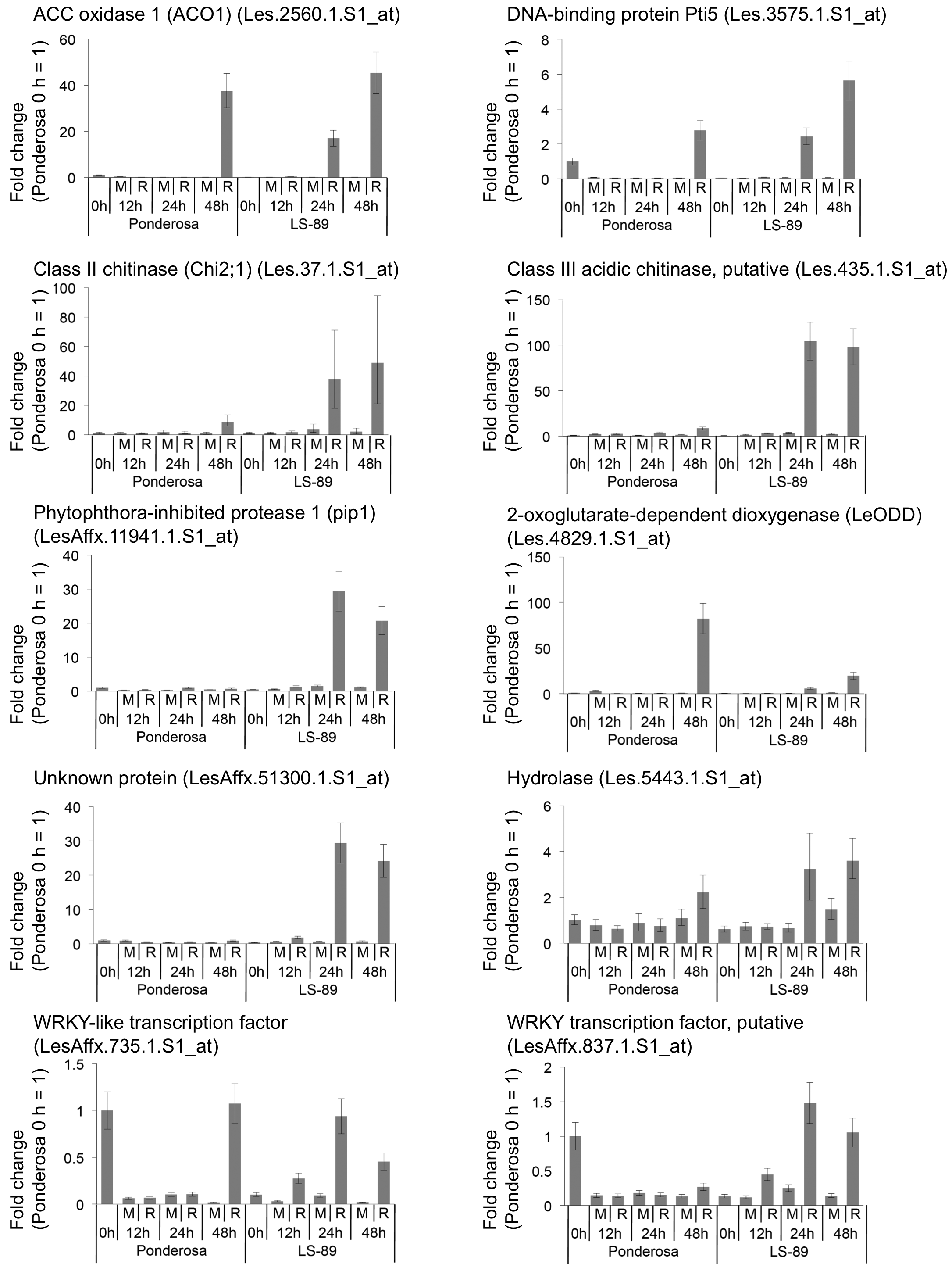

Supplement: Figure S1 — Real-time quantitative RT-PCR analysis of time course of relative transcription levels of genes in resistant tomato cv. LS-89 and susceptible tomato cv. Ponderosa after inoculation with Ralstonia solanacearum (R) or water (mock, M). Genes: ACC oxidase 1 (ACO1), DNA binding protein Pti5, two chitinases, phytophthora-inhibited protease1 (pip1), 2-oxoglutarate-dependent dioxygenase (LeODD), unknown protein (LesAffx.51300.1.S1_at), hydrolase (Les.5443.1.S1_at) and two WRKY transcription factors. Total RNA was extracted from stems to analyze expression at 0, 12, 24 and 48 hpi. Sample from Ponderosa at 0 hpi was used for calibration. (TIF) [file pone.0046763.s001.tif]

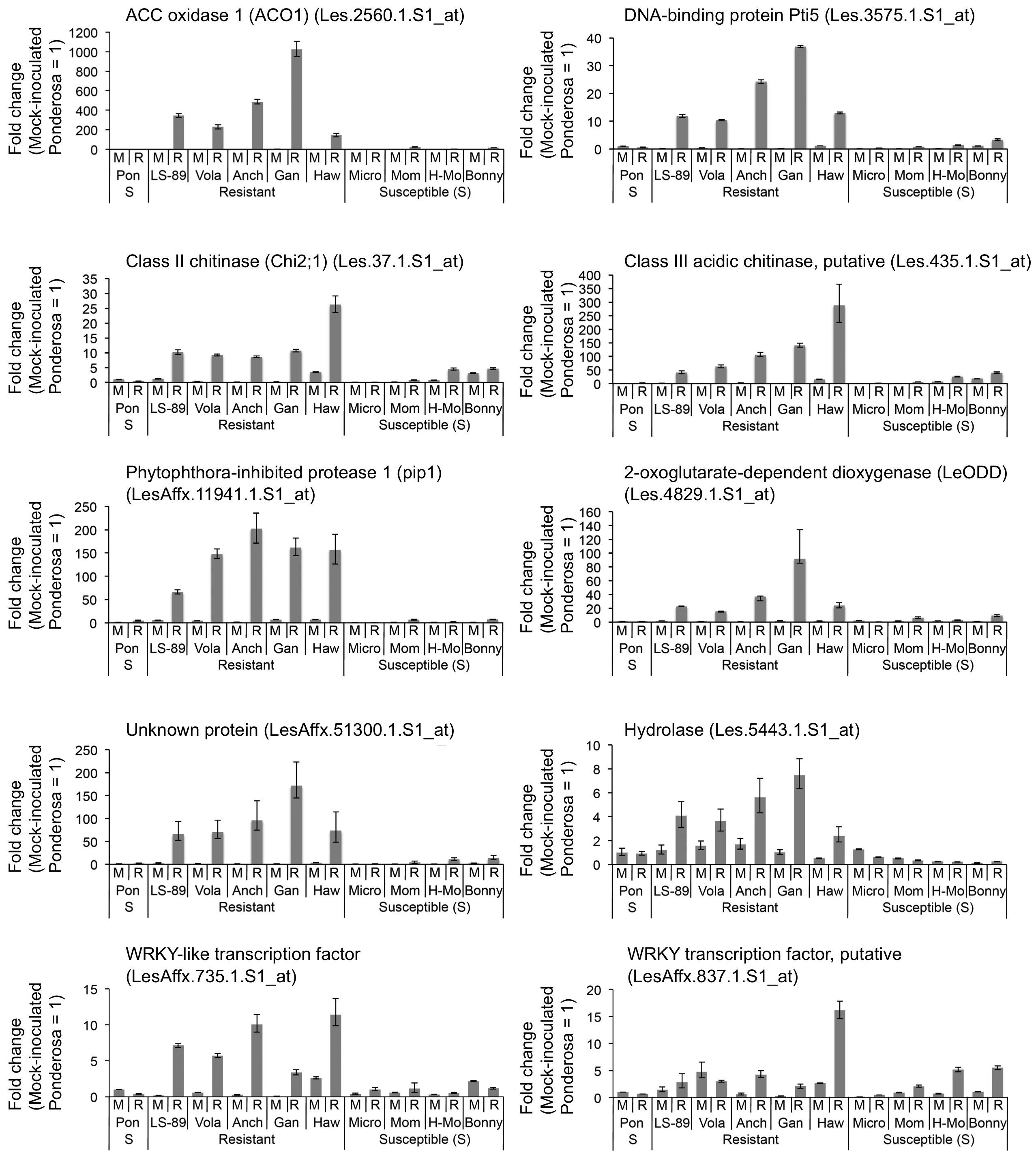

Supplement: Figure S2 — Real-time quantitative RT-PCR to assess relative transcription levels of genes in stems of various tomato cultivars at 1 dpi with water (mock, M) or Ralstonia solanacearum (R). Genes: ACC oxidase 1 (ACO1), DNA binding protein Pti5, two chitinases, phytophthora-inhibited protease1 (pip1), 2-oxoglutarate-dependent dioxygenase (LeODD), unknown protein (LesAffx.51300.1.S1_at), hydrolase (Les.5443.1.S1_at) and two WRKY transcription factors. Resistant cultivars: LS-89, Volante (Vola), Anchor T (Anch), Ganbarune (Gan) and Hawaii7996 (Haw). Susceptible cultivars: Ponderosa (Pon), Micro-Tom (Micro), Momotaro (Mom), House-Momotaro (H-Mo) and Bonny Best (Bonny). Sample from mock-inoculated Ponderosa stems was used for calibration. (TIF) [file pone.0046763.s002.tif]
