# Supplementary material for: Transcriptome Analysis of Quantitative Resistance-Specific Response upon Ralstonia solanacearum Infection in Tomato
Source: PLoS One. 2012 Oct 5;7(10):e46763. doi: 10.1371/journal.pone.0046763 (PMC3465262; doi:10.1371/journal.pone.0046763)
Supplement: Text S1 — Primer pairs for real-time quantitative RT-PCR in Figures S1 and S2. (DOC) [file pone.0046763.s004.doc]

**Supporting information**

**Text S1**

**Primer pairs for real-time quantitative RT-PCR in Figures S1 and S2**

ACC oxidase 1 (ACO1) (Les.2560.1.S1_at)

Forward primer (Les2560-F) TGCACAAACAGACGGGACACGA

Reverse primer (Les2560-R) TTGGCTTGAAACTTGAGTCCAGCA

DNA-binding protein Pti5 (Les.3575.1.S1_at)

Forward primer (Pti5-F) CGACGTCGTCCGTGGGGGAA

Reverse primer (Pti5-R) AGTAGTGCCTTAGCACCTCGCA

Class II chitinase (Chi2;1) (Les.37.1.S1_at)

Forward primer (Chitinase37-F) TGGGGCAGCAGGGAGTGCAA

Reverse primer (Chitinase37-R) TACACCAAAGCCCGGGACGC

Class III acidic chitinase, putative (Les.435.1.S1_at)

Forward primer (Les435-F) TGCAGCTCCACAATGTCCATTCCC

Reverse primer (Les435-R) TGGAGCGGCGGGCAATCCTA

Phytophthora-inhibited protease 1(pip1) (LesAffx.11941.1.S1_at)

Forward primer (pip1-F) AGAGGGAGTGTCACAGGAGTCAAGG

Reverse primer (pip1-R) TGTCTGCGTAGAGCAATCCAATAGTGT

2-oxoglutarate-dependent dioxygenase (LeODD) (Les.4829.1.S1_at)

Forward primer (LeODD-F) TGCTGAGGGACTAGGCATTTTGTGC

Reverse primer (LeODD-R) CCATGAGTAGGAGGAACATCAACCCA

Unknown protein (LesAffx.51300.1.S1_at)

Forward primer (Affx51300-F) ACGGTCAGAAAGATCGAACCAAGGC

Reverse primer (Affx51300-R) GCCGGGCCAACGCTAACCAA

Hydrolase (Les.5443.1.S1_at)

Forward primer (L5443-F) GGCTCCAGGAAACTCAATGTCAAGC

Reverse primer (L5443-R) ACCGTAACTCCCAGGTACTCATGGA

WRKY-like transcription factor (LesAffx.735.1.S1_at)

Forward primer (WRKY-LesAffx735-F) AGAGAACAGAAGGCGGAAGATGGA

Reverse primer (WRKY-LesAffx735-R) CGCGTTTGGCTGGTTTGTTACATCC

WRKY transcription factor, putative (LesAffx.837.1.S1_at)

Forward primer (WRKY-LesAffx837-F) ACGATCAAGAATGCCGGGCCA

Reverse primer (WRKY-LesAffx837-R) TGAGCAGAGGGAGAAGAAGGCA
